# A comparative study of canine epididymal sperm collection techniques and cryopreservation

**DOI:** 10.3389/fvets.2023.1181054

**Published:** 2023-10-24

**Authors:** Hiba Ali Hassan, Penelope Banchi, Guillaume Domain, Rosemary El Khoury, Rana Chaaya, Eline Wydooghe, Katrien Smits, Ann Van Soom

**Affiliations:** ^1^Reproductive Biology Unit, Faculty of Veterinary Medicine, Department of Internal Medicine, Reproduction and Population Medicine, Ghent University, Merelbeke, Belgium; ^2^Department of Veterinary Sciences, Faculty of Veterinary Medicine, University of Turin, Grugliasco, Italy; ^3^Faculty of Agronomy and Veterinary Medicine, Department of Veterinary Medicine, Lebanese University, Dekwaneh, Lebanon; ^4^Centre of Expertise for Agro- & Biotechnology, Department of Biotechnology, VIVES University College, Roeselare, Belgium

**Keywords:** dog, epididymal spermatozoa, collection method, epididymal mincing, single incision epididymal sperm aspiration, erythrocyte lysis buffer

## Abstract

**Introduction:**

An optimized collection method and freezing protocol for preservation of epididymal spermatozoa remains a topic of interest to many scientists. The current study focused on the collection and preservation of canine epididymal spermatozoa. During the process of collection of canine epididymal spermatozoa, blood content can occur, which may affect sperm cryopreservation in a negative way. Here, we compared first two epididymal sperm collection techniques [epididymal mincing (EM) and single incision epididymal sperm aspiration (SESA)]; and next we tried to solve the issue of blood content using an erythrocyte lysis buffer (ELB).

**Methods:**

Hence spermatozoa were collected after weighing the epididymides, either by EM or SESA, and sperm quality assessed prior to and post freezing (concentration, total sperm output (TSO), motility, viability and morphology). Next, new sperm samples were collected from eight epididymides by EM and subjected either to a standard freezing protocol or to an ELB treatment freezing protocol. Post-thaw sperm parameters (concentration, TSO, motility, viability and morphology), including intracellular reactive oxygen species (ROS) and lipid peroxidation were assessed. The correlation between the weight of the epididymis and the TSO was evaluated based on the collection technique, and differences in sperm parameters were detected both within different collection techniques and between different pre-freezing treatment protocols.

**Results:**

There was a very strong correlation between the weight of the epididymis and the TSO for the EM technique (*p * =  0.002, R^2^ =  0.6), along with an increased sperm motility with EM compared to SESA (median 80%, inter-quartile range (IQR) 88–65 and median 67.5%, IQR 72.5–52.5, respectively; (*p * =  0.002). Post-thaw samples subjected to ELB treatment freezing protocol had lower motility and higher intracellular ROS compared to the standard freezing protocol (motility: median 56.25%, IQR 60–48.75 and median 70%, IQR 72.5–63, respectively; *p *  =  0.01; ROS: median 78.5%, IQR 81.25–75.5 and median 70%, IQR 70.5–68.75, respectively; (*p * =  0.04).

**Discussion:**

The results indicated that EM is a better technique to harvest epididymal spermatozoa despite the presence of some blood content. Furthermore, the ELB treatment should not be implemented to remove those red blood cells prior to cryopreservation of epididymal spermatozoa in dogs.

## 1. Introduction

Among several factors affecting semen preservation, both collection method and contamination with bacteria, red blood cells, or leukocytes are known to play a main role in the final outcome (1). Different techniques have been described for the collection of ejaculated and epididymal spermatozoa in dogs (1–5). However, when the collection of a full ejaculate is not possible, the cauda of the epididymis represents a key source of mature spermatozoa and, in most cases, the last chance for reproduction in endangered or dead animals. Various methods have been described to retrieve epididymal sperm (6). These include, amongst others, the retrograde wash method (flushing), single-incision epididymal sperm aspiration (SESA) and epididymal mincing (EM) (2, 3, 7). The retrograde wash method is performed by inserting a 24-gage needle into the vas deferens and by flushing the content out of the duct with semen extender or air (3). This method has been shown to be effective in several studies conducted on dogs (2, 3, 7). It was proven to yield a higher sperm recovery than SESA (7) or EM (3). The flushing method, although effective, cannot be used if the vas deferens is shortened during an orchiectomy or if the vas deferens or epididymal duct is damaged during blood vessel removal from the epididymal surface (3). In a study comparing different collection methods of dog epididymal spermatozoa, a faulty catheterization of the ductus deferens was recorded in one out of ten dogs involved in the study, thus alternative techniques must be available (3).

Epididymal mincing has been suggested as a backup option when the flushing method is not feasible (3). A major issue when employing epididymal mincing is contamination by erythrocytes, as hemospermia negatively affects the structure and function of spermatozoa after thawing (1). Specifically, Rijsselaere et al. (8) showed that blood admixture of more than 4% resulted in a drop of post-thaw sperm parameters such as sperm motility, membrane integrity and acrosomal status; in contrast to chilled canine semen, preserved at 4°C, where the addition of blood up to 10% did not have a detrimental effect on sperm functional characteristics. Freezing and thawing of canine hematospermic samples resulted in around 58% of erythrocyte hemolysis, which in turn led to the release of hemoglobin from the ruptured erythrocytes. Furthermore, recent studies have demonstrated that hemoglobin has both direct cytotoxic effects and plays a significant role in the production of harmful reactive oxygen species (ROS), which can cause indirect negative effects (8). In addition, blood content can interfere with analytical devices such as computer-assisted sperm analysis (CASA) during semen quality evaluation. Therefore, the elimination of erythrocytes prior to freezing could be an essential step to improve post-thaw semen quality after EM, especially in highly contaminated samples. However, simple centrifugation will spin down both spermatozoa and erythrocytes in the pellet; and for this reason, scientists investigated different techniques to remove erythrocytes (9). Among these techniques, gradient centrifugation has been proven to efficiently separate viable motile spermatozoa from dead immotile spermatozoa and erythrocytes, but it negatively affects the yield of viable motile spermatozoa (10). Even though low sperm recovery rate could be counteracted by multiple semen collections when ejaculated semen is considered, this effect represents a relevant down-side when dealing with epididymal spermatozoa, which can only be collected once. Therefore, a protocol ensuring no or a limited loss of spermatozoa during sperm-erythrocytes separation is needed. Verheyen et al. (11) were the first to describe the use of an erythrocyte lysis buffer (ELB) in human hematospermic samples and tested its toxicity on capacitated donor spermatozoa. The results showed that after a 5-min exposure of spermatozoa to ELB, the subsequent motility and vitality of the spermatozoa were not affected in a 48-h sperm survival test (10). Since then, ELB medium is routinely used in human reproduction for the preparation of testicular specimen for intracytoplasmic sperm injection (9), and to our knowledge, the effect of ELB has not been tested yet on dog semen. If it could be used to remove blood content prior to freezing of epididymal spermatozoa, this would be highly desirable.

Single-incision epididymal sperm aspiration (SESA), on the other hand, is considered an ideal method for epididymal sperm recovery as it can operate independently of limitations such as ductus deferens obstruction and contamination. For this technique, the epididymis is cut, rather than chopped into small pieces, with a single incision during which the flowing epididymal fluid and spermatozoa are collected. Comparison of EM and SESA has not been performed yet in dogs.

The aim of the present research was therefore (a) to compare the SESA and EM techniques for the recovering of epididymal spermatozoa and (b) to investigate whether the treatment of EM-harvested epididymal spermatozoa with an erythrocyte lysis buffer prior to freezing would improve post-thaw sperm parameters.

## 2. Materials and methods

All products were purchased from Sigma (Sigma, St. Louis, United States) except if stated otherwise.

### 2.1. Comparison of collection methods

#### 2.1.1. Sample collection

A total of fifteen healthy dogs aged from one to six years old of different breeds and body weights were presented for routine orchiectomy at veterinary clinics across Beirut, Lebanon between January 2021 and May 2021. Immediately following orchiectomy, testes and epididymides were placed in a holding medium consisting of 0.9% physiological saline solution supplemented with gentamycin (0.1 mg/mL) (12), kept cool in a portable fridge (4°C), and transported to the laboratory where they were processed within 24 h of collection. Prior to sperm collection, testes were removed from the holding medium, washed with Dulbecco’s phosphate-buffered saline (DPBS) and carefully wiped dry using an aseptic gauze. Each epididymis, along with the vas deferens, was then carefully dissected away from the testis, weighed on a precise balance (BS 300, PCE instrument, United Kingdom), and epididymal spermatozoa were extracted by EM or by SESA. Exclusion criteria were TSO of lower than 200×10^6^ spermatozoa after collection.

For the EM method, the cauda epididymides along with the vas deferens were placed in a petri dish containing 7 mL of DPBS. The spermatozoa present in the ductus deferens were squeezed out into the petri dish and the cauda of the epididymis was subjected to mincing, using a scalpel blade, to allow spermatozoa to swim out of the cauda (2). After 10 min of incubation at 37°C, the medium containing the spermatozoa was collected using an automatic pipette and placed into a 15 mL falcon tube.

For the SESA method, the cauda epididymis was fixed by a homeostatic forceps. Using a scalpel blade, a single incision was made on the cauda epididymis, taking care to avoid blood vessels. The flowing epididymal fluid and sperm were then collected with an automatic pipette and placed into a 15 mL falcon tube containing DPBS (7). The spermatozoa present in the ductus deferens were squeezed out directly into the same tube.

#### 2.1.2. Sperm quality assessment

Sperm concentration was determined using a Bürker counting chamber and was used to calculate total spermatozoa output (TSO) based on the sample volume.

Sperm motility was assessed subjectively under a phase-contrast microscope (i4 Infinity, LW Scientific, United States) equipped with a warming stage at 37°C. Two equally experienced operators blindly evaluated all samples, and the mean was calculated and recorded.

Viability and morphology of spermatozoa were assessed on eosin/nigrosin stained smears under a light microscope (Olympus CX23, Tokyo, Japan) at 1000 x magnification under oil immersion. Two hundred spermatozoa were counted and classified as alive/dead, and the percentages of normal and abnormal (abnormal head, abnormal tail, proximal cytoplasmic droplet, and distal cytoplasmic droplet) spermatozoa were recorded.

#### 2.1.3. Cryopreservation and thawing

All samples were centrifuged at 720 x *g* for 5 min at 22°C (13) to discard the supernatant and resuspend the sperm pellet into freezing extender I (TRIS-citric acid-glucose-based extender containing 20% of egg yolk), containing 3% glycerol (Uppsala I), until a concentration of 400 × 10^6^ spermatozoa/mL was reached (14). Extended samples were then slowly cooled to 4°C for 90 min and semen extender II, containing 7% glycerol and 1% Equex STM paste (Uppsala II), was added to reach a concentration of 200 × 10^6^ spermatozoa/mL just before cryopreservation (14). Samples were loaded into 0.5 mL straws, placed 5 cm above the surface of liquid nitrogen for 10 min, and finally plunged into liquid nitrogen where they were stored for at least 1 week (15).

Thawing was obtained by submerging the straws in a 37°C warm water bath for 30 s (16). Semen quality was then evaluated after 5 min incubation at 37°C for the same sperm parameters as before cryopreservation.

### 2.2. Cryopreservation of epididymal spermatozoa with and without erythrocyte lysis buffer

#### 2.2.1. Sample collection

Eight epididymides from dogs aged more than 1 year old of different breeds and body weights were presented for routine orchiectomy at different veterinary clinics across Beirut, Lebanon between June 2021 and September 2021. Based on the previous results, epididymal spermatozoa from each epididymis were collected by EM, as described earlier, and placed into 15 mL falcon tubes. Sperm motility, viability, and morphology were assessed post collection, as described before. Samples were then divided into two equal aliquots and two different freezing protocols were applied on each of the eight samples; standard freezing protocol (control group; as described for 2.1.1. Sample collection) and ELB treatment protocol (treatment group).

Both samples were centrifuged at 720x *g* for 5 min at 22°C, after which the supernatant was removed, and the sperm pellet was either resuspended with semen extender I and cooled to 4°C for 90 min (standard freezing protocol) or with 2 mL of ELB (10X RBC lysis buffer Multi-species, ThermoFisher Scientific, United States). The ELB samples were then centrifuged at 25.76 x *g* for 5 min at 22°C and the pellet was resuspended with 2 mL DPBS and centrifuged at 25.76 x *g* for 10 min. After removal of the supernatant, the pellet was resuspended with semen extender I and cooled to 4°C for 90 min. Both groups where then further processed for cryopreservation as described in experiment I.

#### 2.2.2. Sperm quality assessment

Sperm motility, viability, morphology were assessed on post-thaw samples, as described before. Intracellular reactive oxygen species (ROS) were determined with Nitroblue Tetrazolium (NBT) according to Raad et al. (17). Nitroblue Tetrazolium is a yellow water-soluble nitro-substituted aromatic tetrazolium compound. It is a histochemical method that detects the cellular origin of ROS, in this case spermatozoa. Nitroblue Tetrazolium reacts with intracellular superoxide ions to form a blue-black formazan in the cytoplasm of the sperm cell (18). Thawed extended spermatozoa were first washed twice with DPBS to remove the extender. A 0.1% NBT solution (v:v) was added to non-extended spermatozoa and incubated at 37°C for 30 min. The mixture was centrifuged at 250 x *g* for 5 min and the pellet was smeared onto slides. The air-dried slide was then stained with Wright’s eosin methylene blue (Ref.45253, Sigma, St. Louis, United States) and observed under light microscope (Olympus CX23, Tokyo, Japan) at 100 x magnification under oil immersion. Two hundred spermatozoa were counted and classified as NBT-positive or NBT-negative (17). Spermatozoa were considered NBT-positive when blue-black formazan was detected.

A thiobarbituric acid reactive substances (TBARS) test was performed to assess the resistance of spermatozoa to oxidative stress. Specifically, this assay measures the level of malondialdehyde (MDA), an end-product of lipid peroxidation, produced after challenging spermatozoa to a ROS generating environment. Malondialdehyde reacts with thiobarbituric acid (TBA), generating a colorimetric reaction that can be detected by spectrophotometry. Briefly, 200 μL of washed spermatozoa was incubated with 50 μL of 4 mM ferrous sulfate and 50 μL of 20 mM sodium ascorbate at 37°C for 90 min in the dark. Then, 600 μL of 10% trichloroacetic acid at 4°C was added and the sample was centrifuged at 21.13 x *g* at 4°C for 15 min. After centrifugation, 500 μL of the supernatant was mixed with 500 μL of TBA and incubated at 100°C for 15 min. The reaction was stopped by cooling the sample into an ice bath, and the TBARS concentration was measured using a spectrophotometer (Multiskan GO, Thermo Fisher Scientific, United States) at a wavelength of 532 nm (19). The result was expressed as nanograms of TBARS per million spermatozoa. This assay was performed in triplicates for each sample and the mean was calculated.

## 3. Statistical analysis

Statistical analysis was performed using R 4.1.2 (R Inc., Boston, MA, United States). Normality of data distribution was assessed by Shapiro–Wilk tests (*p* < 0.05) and nonparametric statistical analysis was performed. Specifically, Spearman’s rank test was used to assess the correlation between the weight of the epididymis and the TSO for each collection technique. The strength of the correlation was considered according to Leclezio et al. (20): less than 0.2 negligible, 0.2 to 0.29 weak, 0.3 to 0.39 moderate, 0.4 to 0.69 strong, and greater than 0.7 very strong. The effect of the collection technique on pre-freezing and post-thaw sperm parameters were assessed using Mann–Whitney U test. Similarly, the effect of the two different freezing protocols (ELB freezing protocol and standard freezing protocol) was assessed using Mann–Whitney U test. Statistical differences between groups were considered at *p* ≤ 0.05.

## 4. Results

### 4.1. Comparison of collection methods

A very strong correlation between the weight of the epididymis and the TSO was found for the EM technique (*p* = 0.002, Spearman’s correlation coefficient (ρ) = 0.73, R^2^ = 0.6), but not for SESA technique (*p* = 0.18, ρ = 0.36, R^2^ = 0.21). In addition, EM yielded more spermatozoa per epididymis than the SESA technique (median 1.23 ×10^9^ spermatozoa/epididymis, IQR 18.0–9.5 and median 0.70 ×10^9^ spermatozoa/epididymis, IQR 10.38–4.70, respectively; *p* = 0.01).

**Figure 1:**
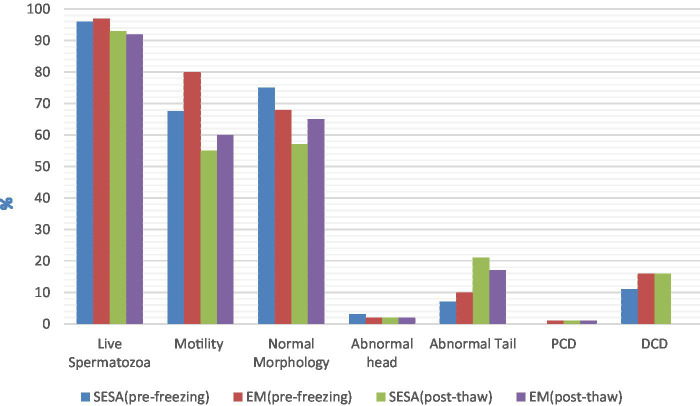
Pre-freezing and post-thaw sperm parameters depending on the collection technique (EM, epididymal mincing; SESA, single incision epididymal sperm aspiration; PCD, proximal cytoplasmic droplet; DCD, distal cytoplasmic droplet).

When comparing fresh sperm parameters, spermatozoa collected by EM had better motility (median 80.0%, IQR 88.0–65.0 and median 67.50%, IQR 72.5–52.5, respectively; *p* = 0.02). No significant differences were found for the other fresh semen parameters investigated, nor for any post-thaw parameters (*p* > 0.05) (Figure 1). In EM, the presence of fresh blood, which was identified by its red color on macroscopic examination, was reported as being consistently prevalent (+++), while it was not detected (−) in SESA.

### 4.2. Cryopreservation of epididymal spermatozoa with and without erythrocyte lysis buffer

Samples cryopreserved with the ELB freezing protocol had a lower motility than samples cryopreserved with the standard freezing protocol (median 56.2%, IQR 60.0–48.7 and median 70.0%, IQR 72.5–63.1, respectively; *p* = 0.01). In addition, intracellular ROS was higher in samples cryopreserved with the ELB freezing protocol compared to the standard freezing protocol (median 78.5%, IQR 81.2–75.5 and median 70.0% IQR 70.5–68.7, respectively; *p* = 0.04). No significant differences were found for the other post-thaw parameters (*p* > 0.05) (Table 1).

**Table 1:** Post-thaw sperm parameters of blood-contaminated epididymal samples subjected to standard freezing protocol or erythrocyte lysis buffer (ELB) freezing protocol.

Parameter	Standard freezing protocol	ELB freezing protocol	*p*-value
Living spermatozoa (%)	97.5 (98.0–96.0)	97.5 (99.0–96.7)	0.48
Motile spermatozoa (%)	70.0 (72.5–63.1)	56.2 (60.0–48.7)	0.01*
Morphologically normal spermatozoa (%)	75.0 (79.0–72.5)	76.5 (78.0–72.7)	0.98
Abnormal sperm heads (%)	1.5 (2.0–1.0)	1.0 (1.0–0.0)	0.10
Abnormal sperm tails (%)	15.5 (17.0–12.7)	13.0 (17.0–11.7)	0.75
Proximal cytoplasmic droplets (%)	6.0 (7.2–4.7)	5.5 (7.7–4.0)	0.90
Distal cytoplasmic droplets (%)	1.0 (2.2–0.7)	2.0 (2.2–0.7)	0.42
Intracellular ROS (%)	70.0 (70.5–68.7)	78.5 (81.2–75.5)	0.04*
Lipid peroxidation (ng TBARS/10^6^spz)	4.3 (5.7–2.6)	3.6 (5.6–2.5)	0.96

*The difference is statistically significant; data presented as Median (IQR, interquartile range).

## 5. Discussion

The current study provides evidence that epididymal sperm retrieval and quality is more efficient with the EM technique in comparison to the SESA technique in the dog. However, the downside of blood content of the sample, inevitably present when the EM technique is applied, could not be solved by employing the ELB freezing protocol without affecting sperm parameters.

When collecting epididymal spermatozoa, the aim is to maximize sperm recovery as this is often the last attempt to preserve gametes from an individual. The median sperm count and post-thaw sperm motility were 1.23 × 10^9^ spermatozoa and 60% for EM and 0.70 × 10^9^ spermatozoa and 55% for SESA, respectively. Therefore, both techniques yielded sufficient spermatozoa of good quality for a later application in assisted reproductive technologies, especially since intrauterine insemination with frozen epididymal spermatozoa, whose post-thaw motility ranged from 20 to 30%, resulted in pregnancies (80% conception rate) and delivery of puppies (Mean ± SE: 2.3 ± 0.9) (21). The difference in sperm recovery between the two techniques may be explained by the way spermatozoa are collected. While spermatozoa flow out of the incision made in the epididymides with the SESA technique, they are given the time to swim up in the collection medium during the incubation period following EM, leading to the recovery of more spermatozoa. The difference in sperm motility can also be related to the method of collection as epididymal sperm parameters have been shown to be similar within paired epididymides (22). Varesi et al. (2) also demonstrated that sperm collected via percutaneous epididymal sperm aspiration had a decreased, although non-significant, motility compared to the mincing method. This discrepancy in motility may be attributed to the prolonged incubation time at 37°C in the mincing technique, which may have allowed the activation of previously dormant sperm cells (23). Our results revealed no differences between the two collection methods with regard to pre-freezing sperm morphology, nor for all post-thaw sperm parameters. As the spermatozoa by both methods were obtained from the same animal, it was anticipated that the collection method would yield comparable sperm morphology.

**Figure 2:**
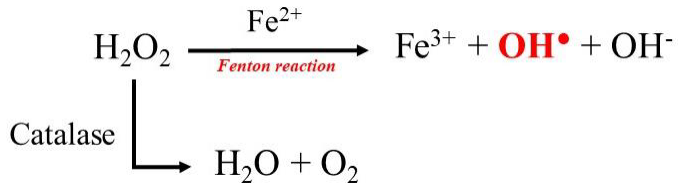
Interplay between Fenton reaction and Catalase action.

Sperm motility was assessed subjectively in most studies involving EM, as the presence of erythrocytes may lead to unreliable values when a CASA device is used. Erythrocytes may indeed be identified as non-motile sperm, resulting in an overstated sperm count and an understated sperm motility (24). The possibility of eliminating red blood cells or the use of software capable of differentiating sperm cells from other cells would therefore provide an objective assessment of sperm motility in future experiments. It is also undeniable that blood content is a relevant down-side of the EM technique as the cryopreservation of hematospermic samples results in a decreased sperm motility, plasma membrane integrity, and acrosome integrity after thawing (8). This lower freezability is thought to be the result of the hemoglobin released from the erythrocytes during the freezing–thawing process (8). Hemoglobin is a rich source of heme molecules, which contain iron, a potentially toxic substance (8). Under normal circumstances, the iron ion in hemoglobin is in the ferrous (Fe^2+^) state, bound to a histidine residue and coordinated to the heme porphyrin ring through four nitrogen atoms (25). When Fe^2+^ reacts with hydrogen peroxide through a process called the Fenton reaction, hydroxyl radicals (OH^●^) are formed, which are the most unstable form of reactive oxygen species (26) (Figure 2). The enzymatic antioxidant catalase can prevent the formation of hydroxyl radicals by catalyzing the breakdown of hydrogen peroxide into water and oxygen (26). However, catalase is not detectable in the epididymis of dogs (26), which suggests that hydroxyl radicals are overproduced as a result.

Therefore, preservation protocols including the removal of erythrocytes prior to freezing are needed. When using density gradient centrifugation, result showed a loss of spermatozoa within a 51%-to-64% interval in ejaculated canine semen (19). As each good quality spermatozoon is essential in case of epididymal spermatozoa, other approaches must be considered to remove cellular contaminants.

The use of a ELB prior to sperm cryopreservation was investigated in this study but this treatment affected post-thaw sperm motility and increased post-thaw intracellular ROS production. The effect on sperm motility may confirm the negative effect that hemoglobin exerts on spermatozoa but is inconsistent with the finding of Soygur et al. (27) who described no effect of ELB on post-thaw sperm motility in humans. Another study on human sperm showed a decrease in sperm motility, viability, and an increase in DNA fragmentation after treatment with ELB (28), but the exposure time of the lysis buffer to spermatozoa was longer in the latter study and we used an exposure time of 5 to 10 s, which was similar to Soygur et al. (27). The decrease in motility could be related to the composition of the lysis buffer. Allahkarami et al. (29) reported a significant negative correlation between seminal plasma ammonium levels and sperm motility, with ammonium being a critical component of ELB. Also, samples treated with ELB were subjected to two additional centrifugation steps at low g-force, compared to untreated samples. Specifically, the function and motility of spermatozoa are known to be sensitive to centrifugal force in other species (30, 31) In dogs, motility of chilled sperm will decrease significantly over time but is not affected by centrifugal force, at least until 2,880 x *g* (13). Even though this suggests the insensitivity of canine semen to centrifugation, in this study, the semen was stored at 4°C and not cryopreserved (13). Thus, the possible damage of the centrifugation process to the spermatozoa after cryopreservation was not assessed.

Spermatozoa from samples treated with the ELB exhibited significantly more intracellular ROS than spermatozoa from untreated samples. Reactive oxygen species can be produced by the spermatozoa either at the level of the plasma membrane and/or at the level of mitochondria (18). The study found that the ELB treatment might negatively impact mitochondrial activity and potential in sperm, affecting motility. However, it did not induce increased oxidative stress to spermatozoa. Both investigated sperm collection methods (EM and SESA) showed high cryopreservation potential, with epididymal mincing being superior in recovery and motility but prone to blood content. Single incision epididymal sperm aspiration, yielding uncontaminated sperm with basic equipment and no specialized training, may be significant. The study discourages ELB use for epididymal sperm intended for cryopreservation and suggests exploring other approaches like microfluidic sperm separation devices to target future research.

## Data availability statement

The raw data supporting the conclusions of this article will be made available by the authors, without undue reservation.

## Ethics statement

Ethical approval was not required for the study involving animals in accordance with the local legislation and institutional requirements because the samples used in our research were epididymis collected after orchiectomy. The reason for castration was not directly related to our study; however, we utilized these samples for research purposes rather than discarding them.

## Author contributions

HA, EW, and AS: conceptualization. HA and EW: methodology. HA and RK: investigation. PB: statistical analysis. EW and RC: resources. HA: writing – original draft preparation. PB, GD, KS, and AS: writing – review and editing. HA and PB: visualization. EW, RC, and AS: supervision. AS: funding acquisition. All authors contributed to the article and approved the submitted version.
